# Clinical Guideline for Detection and Management of Magnesium Deficiency in Ambulatory Care

**DOI:** 10.3390/nu17050887

**Published:** 2025-02-28

**Authors:** Sherrie Colaneri-Day, Andrea Rosanoff

**Affiliations:** CMER—Center for Magnesium Education & Research, Pahoa, HI 96778, USA; arosanoff@gmail.com

**Keywords:** magnesium deficiency, clinical guideline, ambulatory care, lack of knowledge, chronic disease

## Abstract

*Background:* Magnesium (Mg) deficiency is associated with many common chronic conditions and potentially severe health care outcomes, including cardiovascular disease, cardiovascular risk factors, and diabetes. However, Mg deficiency is underdiagnosed and often underrecognized in the ambulatory health care setting, and nutrition education and training are often limited for health care providers (HCPs). *Methods:* A clinical guideline for detecting and treating Mg deficiency in the ambulatory care setting was developed. A pilot study was conducted in which HCPs received education on Mg and completed pre-test and post-test questionnaires to assess the intervention efficacy of the guideline. *Results:* Ten HCPs participated in the pilot study via telephone or face-to-face session. In general, there was a statistically significant increase in Mg knowledge among HCPs, due to the intervention of presentation of the guideline, with a nonsignificant increase in clinical practice application. However, the 1-month follow-up survey results showed that HCPs were likely to incorporate Mg assessment and treatment tools from the guideline in their future practice. *Conclusions:* These findings suggest that the use of the proposed clinical guideline may increase HCP knowledge and improve the diagnosis and treatment of Mg deficiency. Further use, development, and evaluation of this guideline is warranted.

## 1. Introduction

Magnesium (Mg) is an essential mineral required for numerous physiological functions [1,2]. Chronic low dietary intake of Mg is widespread and is associated with adverse health outcomes such as hypertension, diabetes, osteoporosis, inflammation, cardiovascular diseases, other risk factors, and some cancers [1,2,3,4]. Nutrition education and training (including on Mg) for many health care providers (HCPs), including physicians, nurse practitioners, and physician assistants, among others, is generally limited [5]. Traditional and current HCP training does not incorporate the identification of Mg deficit in the diagnosis of acute and chronic diseases [6]. Schwalfenberg and Genuis [3] reported that trainee programs in physiology, nutrition, and dietetics provide health education on Mg, but discussion of nutritional deficits, including Mg deficiency, is lost in HCP education.

**Figure 1:**
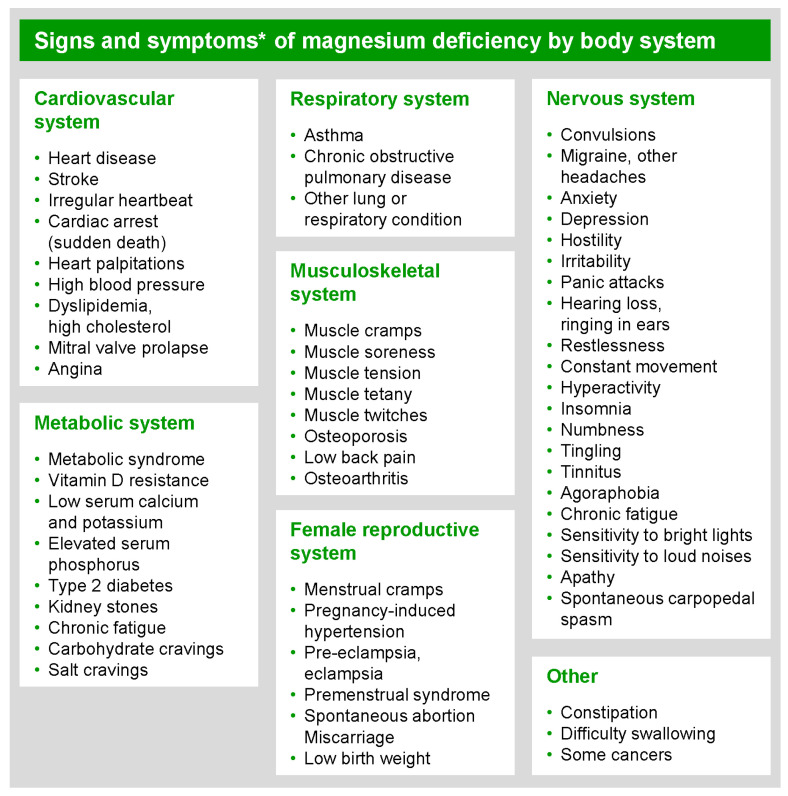
Sign and symptoms associated with magnesium deficit in peer-reviewed literature. * May be nonspecific to magnesium; associated with Mg in peer-reviewed literature. Sources: Data are compiled from Frederickson [11], Seelig and Rosanoff [12], and a CMER compiled database of peer-reviewed Mg research.

Currently, approximately 60% of adults have an inadequate dietary intake of Mg due to common modern food processing [7] and dietary practices, medications, and modern farming and cultivation techniques [8]. Mg deficiency is associated with an increased risk of disease, illness, and complications (e.g., diabetes, hypertension, cardiovascular disorders, and depression), among other conditions [3]. Signs and symptoms of Mg deficiency (see Figure 1) are numerous, nonspecific, and widespread [9]. In addition, clinical diagnosis of Mg deficit in the ambulatory care setting is not straightforward, as multiple serum Mg units as well as reference range values are in common use [10].

Despite the promise of enhanced medical education and training in nutrition to reduce health and economic burdens from cardiovascular diseases a decade ago [13,14], current collaborative nutrition education programs in medical schools show low priority. Less than 22% of surveyed schools meet the minimum recommendation of 25 h of nutrition education for medical students [15]. Specific to nutritional Mg education in medical and nursing schools, previous studies suggest that increasing HCP education in nutrition can potentially improve recognition of Mg deficiency in the ambulatory setting [3,16,17] and addressing clinician awareness of nutritional Mg deficit can improve health care outcomes. Thus, increased awareness of the importance of Mg, prevention and treatment of Mg deficiency, and the role of this mineral in related disorders is needed in the ambulatory setting. Such awareness can be achieved through educational interventions that improve HCP knowledge and skills as well as attitudes regarding competency in that knowledge [18,19]. Targeted interventions for HCPs that use algorithm-based clinical guidelines have been shown to influence provider practice [20]. However, clinical guidelines for the identification and treatment of Mg deficiency in the ambulatory care setting do not exist [21].

This study aimed to improve ambulatory HCP knowledge of Mg deficiency and to promote increased identification and management of Mg deficiency in ambulatory health care through a structured guideline leading to the use of such knowledge in clinical practice. To organize the best available evidence to support clinical decision-making, improve the quality of care, and provide optimal outcomes, a clinical guideline for detecting and treating Mg deficiency in the ambulatory care setting was developed and pilot tested using an educational intervention [21]. The guideline presented here arises from an inter-disciplinary collaboration between an experienced HCP with 28 years of clinical practice in the ambulatory setting and a nutritional Mg researcher with over 30 years of study and publications in the peer-reviewed field of nutritional Mg. Such collaboration is backed by research showing that such inter-disciplinary approaches can enhance nursing school nutrition education [22] and may support growth for the needed role of clinical nurse specialists in nutrition [23].

## 2. Materials and Methods

### 2.1. Guideline Development

A clinical guideline for recognition and treatment of Mg deficiency in ambulatory care (Clinical Guideline for Magnesium Deficiency, referred to hereinafter as the guideline) was developed as an inter-disciplinary collaboration of an experienced HCP and an expert in peer-reviewed literature on nutritional Mg. Information for this guideline was drawn from extensive searches of PubMed, Google Scholar, and Scopus plus full access to a CMER Endnote library on Mg, began in 2002, which is kept current and now has 7756 records dating back to 1910. This core information was gathered and written into a guideline that might be a useful tool for HCPs, prioritizing Mg-associated diseases and symptoms, serum Mg assessment studies and reviews, oral Mg trials (prioritizing meta-analyses when available), and reliable oral Mg doses and serum Mg references ranges appropriate for the ambulatory setting [21]. The complete guideline is presented in Appendix A.

### 2.2. Guideline Pilot Testing

Information from the earliest drafts of this guideline was pilot tested as an educational intervention for a small group of HCPs in an educational program targeted to ambulatory HCPs [21]. The immediate goals were as follows: (1) to see if the presentation of guideline information could improve HCP knowledge regarding Mg deficiency and (2) if such knowledge might influence the incorporation of such knowledge through the adoption of the guideline in clinical practice [20,21,24]. The study was conducted in accordance with the Declaration of Helsinki, and approved by the Institutional Review Board of Keiser University (protocol code IRB000FC19DN15, “Improving Healthcare Providers’ Detection and Management of Magnesium Deficiency Through a Targeted Intervention and Clinical Guideline”, approved 15 December 2019). Informed consent was obtained from all subjects involved in the study.

The guideline information was presented as an educational intervention to HCPs either in a group face-to-face session or individually by telephone. Evaluation of intervention effectiveness in enhancing knowledge of Mg in clinical practice was measured via pre- vs. post-intervention test questionnaires (Supplementary Materials S1) [21]. For the face-to-face and telephone groups, the educational intervention began with the administration of the pre-test questionnaire to assess participant knowledge. The questionnaire included 17 questions (13 on Mg knowledge and 4 on Mg use in clinical practice) and was designed to be scored with a Likert-type scale to assess guideline effectiveness. The lead author (S.C.-D.) of this study gave an oral presentation describing the intervention and shared a poster version; copies were provided to participants. The face-to-face group then completed the post-test questionnaire. The telephone group was provided the pre-test questionnaire verbally, the intervention and guideline were discussed, and then the post-test questionnaire was administered.

Mean ± SD scores were calculated pre-test and post-test for all questions (*N* = 17), for general Mg knowledge questions (*n* = 13), and for clinical practice questions (*n* = 4). Pre-test vs. post-test mean scores for each set of questions were tested for statistical significance using a paired *t* test. Additionally, effect size testing was performed for each set of questions using Cohen’s *D*.

One month later, a 6-question follow-up survey (Supplementary Materials S2) was administered to assess HCP integration of guideline knowledge and actual application to clinical practice [21]. Each question had 4 possible responses (*rarely*, *sometimes*, *most of the time*, or *always*). Numeric score ranges were assigned for each category; mean ± SD scores for 9 of 10 participants (1 was lost to follow-up) were calculated for each question [21].

## 3. Results

The guideline was presented as an educational intervention to ten practitioners. Two HCPs participated in the group face-to-face session and eight participated individually by telephone. Participant demographics are presented in Table 1.

Improvement in Mg knowledge was statistically significant for the 17-question educational intervention (possible score range = 170–510), with mean ± SD pre-test and post-test scores of 431 ± 46.8 and 492 ± 14, respectively (*p* = 0.0018, Table 2). The effect size for the teaching intervention was very large (Cohen’s *D* = 1.77).

The educational intervention significantly increased participants’ general Mg knowledge (questions 1–13; total score range = 130–390), with mean ± SD pre-test and post-test scores of 321 ± 30.3 and 374 ± 11.7, respectively (*p* = 0.00049). The effect size for increased general knowledge was extremely large (Cohen’s *D* = 2.30).

Increased clinical practice application (questions 14–17, possible score range = 40–120) was observed among HCPs but was not statistically significant, with mean ± SD pre-test and post-test scores of 110 ± 24.9 and 118 ± 6.32, respectively (*p* = 0.35). The effect size for clinical practice application was small (Cohen’s *D* = 0.44).

Table 3 presents the responses to the follow-up survey administered 1 month after the intervention. Participants reported that the guideline was *sometimes* used (question 1), and they intended to *always* use the guideline as an at-a-glance tool and laboratory reference (question 2). Participants reported that they *always* found this information helpful (question 3). Participants answered *most of the time* when asked about their intention to use Mg deficiency in their differential diagnosis (question 4), their intention to test for serum Mg (question 5), and whether they are treating their patients for Mg deficiency (question 6). These findings suggest that HCPs were likely to implement Mg assessment in their routine clinical practice after they received the educational intervention.

## 4. Discussion

Despite robust research on the role of Mg in chronic diseases, the importance of Mg for health remains underrecognized due to gaps in knowledge [8,9] and the lack of nutrition education and training for HCPs [5]. The lead author (S.C.-D.) has 28+ years of clinical experience and found that Mg blood testing is ordered notably less frequently in the ambulatory care setting compared with in the hospital. Most current laboratory values for serum Mg reference ranges are not reliable in the ambulatory setting, because patients with Mg deficiency may seem to have normal laboratory values when, in fact, they are deficient because serum Mg falls within traditional normal values [10,25]. Increased awareness is needed among HCPs in terms of Mg research and how this essential mineral affects several common conditions that present in clinical practice [8,26]. The guideline for Mg developed and tested in this study provides a tool to allay this situation [21]. This guideline development focus was on the assessment of CLMD, a low Mg status in ambulatory patients, using serum Mg values plus a list of symptoms and physical examination of the patient. This is certainly a good beginning, as such assessment is so currently underused. However, this guideline is not comprehensive and does not represent all peer-reviewed knowledge of nutritional Mg, so might best be called Mg Guidelines One. The future development of this guideline could include the role of obesity in Mg requirement, the role of calcium ratio with Mg in both dietary and serum assessment, and a comprehensive guide on medication interaction with Mg status, an area of vast misinformation on current social media.

The pilot study potentiated the use of an at-a-glance tool and algorithm-based clinical Mg guideline in modifying clinician practice through increasing knowledge of and recognition of Mg deficiency. In general, there was a significant increase in Mg knowledge among HCPs due to education intervention, but the increase in clinical practice application was not significant. However, HCP responses to the 1-month post-test survey suggest that they are likely to implement the Mg assessment and treatment tools provided in the guideline in their future clinical practice. These findings support the need for education on and application of the guideline as a tool to improve the recognition, diagnosis, and treatment of Mg deficit in the ambulatory setting. Widespread use of this guideline may improve recognition of Mg deficiency in the ambulatory care setting, which may decrease complications related to Mg depletion in common acute and chronic disease states (e.g., cardiac arrhythmia, hypertension, or diabetes, among others).

However, this potential must be further assessed in a larger follow-up study that includes patient outcomes and uses a more rigorous design for effectiveness evaluation. Despite its promising statistical outcome, this pilot study’s small sample size is a grave limitation, as small samples are more prone to errors and results may not be applicable to broader populations of HCPs. Nevertheless, these promising statistical results encouraged us to proceed with developing this much-needed guideline. We urge a full study on this topic, and we have included in the Supplemental Materials our questions/answers and key to scoring to facilitate that purpose. In addition, diverse representation across professional roles, including physicians and nurses, plus a balance of gender groups, needs to be considered for such a future full study in order to broaden the applicability of findings beyond this pilot study, and a formal criteria for question selection, such as the Delphi system [27], could strengthen acceptance of the guideline.

Our limited pilot study did not include a patient outcome measure. Notably, there appeared to be bias in the questions in the clinical segment, as participants seemed to anticipate what the researcher was looking for in terms of the “right answer”. This hindrance was addressed by adding the 1-month follow-up survey, which showed that participants found that the clinical guideline (1) was helpful, (2) was being used, (3) incorporated knowledge of Mg deficiency in the differential diagnosis, (4) increased ordering related to Mg tests in clinical practice, and (5) definitively changed their clinical care. These results showed an inclination among HCPs to change clinical practice, which would not have been noted if this follow-up was not completed.

## 5. Conclusions

This inter-disciplinary collaboration developed and pilot tested a guideline for Mg status detection and treatment for the ambulatory care setting, to the best of our knowledge, the first such published Mg guideline. Although the pilot study population was small, these highly statistically significant findings suggest that the guideline can be useful, and a larger trial is prudent. This study is significant to ambulatory clinical practice because it offers an easy-to-use educational intervention shown to increase HCP knowledge of Mg deficiency, promotes the diagnosis and treatment of Mg deficit in various specialty ambulatory care practice settings, and provides research-updated laboratory values for serum Mg reference ranges germane to the ambulatory care setting.

This study opens to a broader goal: increasing Mg knowledge among clinicians so they can share it with patients. Clinicians should educate patients on and increase their awareness of low Mg in the modern diet, possible effects of medication use on Mg status, and association of low Mg status and immunity on specific disease processes commonly addressed in health care today. Additionally, tools attained by clinicians can improve Mg status monitoring and progress of patients undertaking dietary management, supplementation, and routine testing, which is currently more common in the hospital setting versus the ambulatory setting.

Areas for further research on the application of the guideline and tools presented here may include diet, Mg supplementation type and dosage, patient outcomes, and prevention of Mg deficiency. These efforts may potentially reduce complications of acute and chronic conditions associated with Mg deficiency.

## Figures and Tables

**Table 1:** Participant demographics.

Participant ID	Professional Experience, Year	Gender	Degree	Experience with Mg, Year	Presentation Type
A1	1–3	Female	APN/MSN	Do not know	Telephone
B2	1–3	Female	APN/MSN	None	Face-to-face session
C3	5–10	Female	APN/DNP	5–10	Telephone
D4	≥10	Female	APN/DNP	5–10	Telephone
E5	1–3	Female	APN/MSN	1–5	Telephone
F6	≥10	Female	APN/MSN	5–10	Telephone
G7	3–5	Female	APN/MSN	5–10	Telephone
H8	5–10	Female	APN/MSN	5–10	Telephone
I9	≥10	Male	MD	5–10	Face-to-face session
J10	1–3	Female	APN/MSN	1–5	Telephone

Abbreviations: APN, advanced practice nurse; DNP, doctor of nursing practice; MD, doctor of medicine; Mg, magnesium; MSN, master of science in nursing.

**Table 2:** Participant pre-test and post-test scores.

Participant ID	General Mg Knowledge (13 Questions)		Clinical Practice (4 Questions)		Total (17 Questions)	
	Pre-Test	Post-Test	Pre-Test	Post-Test	Pre-Test	Post-Test
A1	340	370	120	120	460	490
B2	340	370	110	120	450	490
C3	260	370	110	100	370	470
D4	320	360	120	120	440	480
E5	350	370	120	120	470	490
F6	320	390	120	120	440	510
G7	300	390	120	120	420	510
H8	290	370	40	120	330	490
I9	360	390	120	120	480	510
J10	330	360	120	120	450	480
Score, mean ± SD	321 ± 30.3	374 ± 11.7	110 ± 24.9	118 ± 6.32	431 ± 46.8	492 ± 14
*p* value		0.00049		0.35		0.0018
Cohen’s *D*		2.30		0.44		1.77

Abbreviation: Mg, magnesium.

**Table 3:** Range of mean scores for 1-month follow-up survey.

Question	Mean Score Range for Each Question (*n* = 9 Respondents)			
	Rarely (10–15)	Sometimes (15.1–24.9)	Most of the Time (25–34.9)	Always (≥35)
1. Have you used the clinical guideline?		**X**		
2. Now that you have the clinical guideline, do you feel you have and/or will continue to use the at-a-glance tool and laboratory reference to identify patients at risk for magnesium deficiency?				**X**
3. Do you find this clinical guideline helpful?				**X**
4. Do you include magnesium deficiency in your differential diagnosis?			**X**	
5. Do you test or intend to test for magnesium deficiency?			**X**	
6. Are you treating patients for magnesium deficiency?			**X**	

## Data Availability

The original contributions presented in this study are included in the article/Supplementary Material. Further inquiries can be directed to the corresponding author(s).

## Supplementary Materials

The following supporting information can be downloaded at: https://www.mdpi.com/article/10.3390/nu17050887/s1, Supplementary Materials S1: Pre-Test and Post-Test Magnesium Questionnaire With Key; Supplementary Materials S2: Post-Intervention 1-Month Follow-Up Survey With Key.

## Appendix A. Clinical Guideline for Magnesium Deficiency in Ambulatory Care

### Appendix A.1. Purpose

This clinical guideline (hereinafter, the *guideline*) describes approaches to diagnostic assessment and clinical treatment of low magnesium (Mg) status (hypomagnesemia) in adults in ambulatory settings.

### Appendix A.2. Definition

#### Appendix A.2.1. Importance of Mg for Health

Mg is a critical mineral in the human body and is involved in 80% of known body cellular metabolic functions [8]. Mg is an essential mineral absorbed in the gastrointestinal tract; once absorbed, Mg is distributed throughout the body. Only very small amounts of Mg are found in the blood, serum, and red blood cells. The remainder is found in soft tissue, muscle, and bone. The bone is the most important area for storage and exchange of Mg [4,8,28,29]. Currently, 60% of US adults have inadequate dietary intake of Mg [8] due to common dietary practices, medications, and modern farming and cultivation techniques. Mg deficiency is associated with an increased risk of disease, illness, and complications (e.g., diabetes, hypertension, cardiovascular disorders with its risk factors, and depression, among other conditions).

#### Appendix A.2.2. Serum Mg Reference Range

For many clinical laboratories, a normal serum Mg reference range of 0.7–1.0 mmol/L (1.4–2.0 mEq/L; 1.7–2.43 mg/dL) is used. However, research suggests that the cutoff of 0.7 mmol/L is too low to adequately diagnose hypomagnesemia in the ambulatory setting, with 0.9 mmol/L (1.8 mEq/L; 2.2 mg/dL) being ideal to rule out Mg deficit as an underlying cause of symptoms or risk of a chronic latent Mg deficit (CLMD) [30].

### Appendix A.3. Suggested Illustrative Criteria for Assessment (At-a-Glance Tool)

#### Appendix A.3.1. Causes of Mg Deficiency

More than 45% of the US population is Mg deficient [8]. Mg deficiency can result from poor mineral intake due to modern diets, but historical farming practices may also play a role. For example, Mg levels in fruits and vegetables have decreased 80%–90% in the last 100 years [8]. More than half of the modern diet (57.5% in the US) consists of processed food [31]. Processing techniques such as grain refining and vegetable cooking can cause up to an 80% loss of Mg content [32]. Beverages such as soft drinks and coffee increase renal excretion and increase the body’s demand for Mg. Common medications like antacids and proton pump inhibitors can decrease gastrointestinal pH. Antibiotics and contraceptives can affect absorption due to a chemical reaction, and diuretics can increase renal excretion [8]. A low-protein diet can affect Mg availability and absorption. Finally, softened or deionized water can contribute to low Mg intakes, contributing to Mg deficiency [33].

#### Appendix A.3.2. Signs and Symptoms of Mg Deficiency

Many signs and symptoms of Mg deficiency are nonspecific. These signs and symptoms include abdominal pain, constipation, angina, arrhythmia, asthma, ataxia, attention-deficit disorder, chronic fatigue, circulatory disturbances (e.g., stroke, cardiac disease, infarction, or arteriosclerosis), cluster headache, confusion, cramps, depression, diabetes mellitus, epilepsy, hypertension, high cholesterol, migraine, anxiety, panic disorder, insomnia, fatigue, dysregulated eating, decreased energy, osteoporosis, Parkinson’s disease, preeclampsia, stress, dependent disorders, tinnitus, tremor, and weakness [30]. The following table presents an at-a-glance tool with suggested criteria for assessment of possible Mg deficiency.

**Table A1:** Suggested Illustrative Criteria for Assessment (At-a-Glance Tool).

Category	Risk Factors	Criteria/Degree of Risk
Disease	Diabetes, hypertension, cardiac disease, stroke, vascular disease	Major
	Osteoporosis, COPD, depression, anxiety	Minor
	Consumption of soda, processed food (e.g., refined flour, deionized water, high fructose corn syrup)	Major
	Coffee, alcohol, protein	Minor
Medication	Diuretics, antacids, proton pump inhibitors	Major
	Oral contraceptives, antibiotics	Major
	Increase blood-level antibiotics	At risk
Clinical	Leg cramps	Major
	Sleep disorders, fibromyalgia, fatigue	Minor
Metabolic status	Metabolic syndrome	Major
	Body mass index > 30 kg/m^2^ (increases Mg requirement)	Major

*Abbreviations:* COPD, chronic obstructive pulmonary disease; Mg, magnesium. *Source:* Adapted from Workinger et al. [8], with data from Boyle et al. [34] and Kieboom et al. [35].

### Appendix A.4. Diagnostic Criteria

#### Appendix A.4.1. Evidence-Based Interval for Serum Total Mg Concentration

Serum total Mg concentration is the foremost and least expensive test currently used by HCPs to assess Mg status. A serum Mg reference range of 0.75–0.95 mmol/L was determined by measuring healthy individuals aged 18–74 years, defined as normal according to the first National Health and Nutrition Examination Survey (1971–1974) [36]. It is important to note that this range may be assumed to include patients who have normal serum Mg but, in fact, may have CLMD [37] at a serum Mg range of 0.75–0.85 mmol/L.

#### Appendix A.4.2. Laboratory Reference for Diagnosis of Mg Deficiency

The following table presents a laboratory reference for diagnosis of Mg deficit using serum Mg values.

**Table A2:** Laboratory reference for diagnosis of Mg deficit (including CLMD) using serum Mg.

Unit/Parameter	Low Normal	Low-Normal	Low-Normal	Mid-Normal	Ideal	High	Symptomatic
mmol/L	<0.6	<0.7	0.75	0.8	0.9	1.0	2–5
mEq/L	<1.2	<1.4	<1.5	1.6	1.8	2.0	4–10
mg/dL	<1.5	<1.7	<1.8	1.9	2.2	2.4	4.9–12
Diagnostic error, % *		90	50	10	1		
**Initiate Mg treatment**	**Yes**	**Yes**	**Yes**	**Yes**	**Goal**		

Abbreviations: CLMD, chronic latent magnesium deficit; Mg, magnesium. * Percentage of patients for whom Mg deficit not diagnosed, possibly erroneously [30]. *Source:* Data are from Rosanoff et al. [10] and Liebscher and Liebscher [30].

### Appendix A.5. Plan

- **Diagnosis:** When a patient presents with possible Mg deficiency-related symptoms (see Table A1), establish a serum Mg baseline with a chemistry profile including renal function, in case oral Mg therapy is indicated. *Note:* Clinical laboratories do not all define “low serum magnesium” with the same reference range [10], and different units expressing serum Mg are used: the most common are mmol/L, mEq/L, and mg/dL. See Table A2 for a laboratory reference.
- **Treatment:** An adequate Mg diet is always recommended. Such diets must include high Mg foods such as whole grains, nuts, seeds, legumes, and green vegetables every day. For a list of specific foods, see Food Sources of Magnesium on the CMER Center for Magnesium Education and Research website (https://magnesiumeducation.com/food-sources-of-magnesium/) (accessed on 20 February 2025). Mg supplementation should be considered if the serum Mg value is <0.9 mmol/L (1.8 mEq/L; 2.2 mg/dL) [30]. Mg supplementation should be considered necessary if the serum Mg value is ≤0.8 mmol/L (1.6 mEq/L; 1.9 mg/dL) [30] (also see references [10,25]).
- **Goal:** The goal of oral Mg therapy is mitigation of Mg deficiency symptoms plus a serum Mg of 0.9 mmol/L (1.8 mEq/L; 2.2 mg/dL) to ensure remaining symptoms are not Mg related. See Table A2 for serum Mg deficient vs. ideal levels.
- **Mg supplementation therapy**:
  - ***Dose:*** Successful repletion therapy usually requires no less than 600 mg Mg/d. For prevention, 350 mg Mg/d is an average. These doses are for elemental Mg. Such doses of elemental Mg are safe [38], but one can decrease or divide the daily dose if not well tolerated.
  - ***Bioavailability:*** The form and dose of Mg supplement is always a question, as several forms of Mg supplements are available. Generally, the more soluble organic forms of Mg supplements have higher bioavailability than inorganic forms [39]. However, most clinical research using Mg supplements has used inorganic forms of Mg. See below.
  - ***Monitoring:*** Recheck laboratory values after 1 month of oral therapy, then check every 3 months thereafter. Therapy should proceed for at least 3 months and then continue with an elemental Mg dose that holds the serum Mg value between 0.85 and 0.95 mmol/L (1.7–1.9 mEq/L; 2.1–2.3 mg/dL) and preferably not lower than 0.9 mmol/L (1.8 mEq/L; 2.2 mg/dL) to lessen future risks of Mg deficiency [10,25,30]. Refer to Table above: Laboratory reference for diagnosis of Mg deficit using serum Mg.
- **Related benefits:** In addition to reducing the risk of major modern chronic diseases, raising serum Mg levels via oral Mg supplementation may enhance sleep, normalize blood pressure, regulate insulin sensitivity, improve glucose control and lipid profile, decrease anxiety and depression, asthma and related conditions, PMS symptoms, fibromyalgia tenderness, and migraine. Oral Mg supplementation has been shown in clinical trials to benefit heart disease, depression, sleep, cholesterol (total, LDL, and HDL), triglycerides, C-reactive protein, anxiety, and headache.
- **Adverse effects and interactions:** Mg supplementation is very safe and generally well tolerated; product quality is important. Side effects may include nausea, vomiting, loose stool, and diarrhea. If side effects occur or are severe, divide the dose to be taken 2 times a day. The 1997 Institute of Medicine suggested that an upper limit of 350 mg Mg/d supplement [40] has been shown to be an underestimation needing reevaluation [38]. If kidney function is adequate (estimated glomerular filtration rate [eGFR] > 60 mL/min/1.73 m^2^), high elemental Mg doses can be safe as well as beneficial. Recent studies show that Mg can improve vascular function, calcification, and mineral metabolism in people with chronic kidney disease [41] and that oral Mg up to 700 mg/d in subjects with a GFR < 60 mL/min/1.73 m^2^ showed no adverse effects [42], see Table A3.

**Table A3:** Forms of Mg for oral supplement therapy that have shown improvement of symptoms, including in clinical trials.

Form of Mg	No. of Clinical Trials in PubMed (on 3 December 2024)
Sulfate	605 (not intravenous)
Aspartate	755
Chloride	352
Oxide	213
Citrate	153
Pidolate	108
Malate	3
l-Threonate	6
Orotate	18
Glycinate	3
Taurate	Not available *

Abbreviations: Mg, magnesium. * These other forms of Mg are generally recognized as safe and may be beneficial, but published peer-reviewed randomized clinical trials on these forms were not available at the time of this writing.
