# Supplementary material for: Clinical Guideline for Detection and Management of Magnesium Deficiency in Ambulatory Care
Source: Nutrients. 2025 Feb 28;17(5):887. doi: 10.3390/nu17050887 (PMC11901669; doi:10.3390/nu17050887)
Supplement: Supplementary file 1 [file nutrients-17-00887-s001.zip › nutrients-3472611-supplementary.pdf]

Supplemental Material

Clinical Guideline for Detection and Management of Magnesium Deficiency in Ambulatory Care

Sherrie Colaneri-Day * and Andrea Rosanoff

CMER—Center for Magnesium Education & Research, Pahoa, HI 96778, USA; arosanoff@gmail.com
* Correspondence: shercday@gmail.com

Appendix S1. Pre-Test and Post-Test Magnesium Questionnaire With Key

Appendix S2. Post-Intervention 1-Month Follow-Up Survey With Key

Appendix S1. Pre-Test and Post-Test Magnesium Questionnaire With Key

General Information

Circle answer.

1. How many years have you been a health care professional (MD, NP, or PA)?
   a. 1–3 years
   b. 3–5 years
   c. 5–10 years
   d. ≥10 years
2. What is your gender?
   a. Female
   b. Male
3. What is your education level?
   a. APN/master's
   b. APN/DNP
   c. MD
   d. DO
4. How long have you worked with patients with magnesium deficiency?
   a. Never
   b. 1–5 years
   c. 5–≥10 years
   d. Don't know

General Magnesium Knowledge

Circle answer.

1. Magnesium is an essential mineral that supports more than fifty functions in the body.
   a. Yes
   b. No
   c. No opinion
2. If serum magnesium laboratory values are in clinical range, I need not worry about magnesium deficiency for my patient.
   a. Yes
   b. No
   c. No opinion
3. A therapeutic dose of magnesium for adults is 600 mg/d.
   a. Yes
   b. No
   c. No opinion
4. Which of the following is a normal daily dosage for prevention of magnesium deficiency?
   a. 350 mg/day
   b. 100 mg/day
   c. 250 mg/day

5. Serum magnesium levels can play a role in chronic diseases.
    a. Yes
    b. No
    c. No opinion
6. The presence of a chronic disease such as diabetes or hypertension would not be a criterion to further assess serum magnesium.
    a. Yes
    b. No
    c. No opinion
7. Food choices can have a major effect on magnesium levels.
    a. Yes
    b. No
    c. No opinion
8. A one-a-day supplement, bottled water, and enriched flour provides adequate magnesium for most people.
    a. Yes
    b. No
    c. No opinion
9. Are patients with magnesium deficiency prone to several chronic diseases?
    a. Yes
    b. No
    c. No opinion
10. Which of the following is not associated with magnesium deficiency?
    a. Hypertension
    b. Diabetes
    c. Hypothyroidism
    d. None of the above
11. Oral magnesium supplements are generally safe.
    a. Yes
    b. No
    c. No opinion
12. Which of the following is a normal serum blood magnesium level?
    a. 0.8 mmol/L
    b. 1.7 mg/dL
    c. 1.2 MEq/L
13. Magnesium is an essential mineral for normal metabolic function.
    a. Yes
    b. No
    c. No opinion

Clinical Practice

Circle one.

1. Assessment of serum magnesium levels and clinical symptoms should be part of my routine clinical management of magnesium deficiency.
   a. Yes
   b. No
   c. No opinion
2. I am likely to modify my clinical practice by screening and treatment for magnesium deficiency.
   a. Yes
   b. No
   c. No opinion
3. Magnesium deficiency is an important phenomenon in clinical practice.
   a. Yes
   b. No
   c. No opinion
4. It is necessary to educate patients on prevention and/or treatment of magnesium deficiency through diet and supplementation.
   a. Yes
   b. No
   c. No opinion

Key to the Pre-Test and Post-Test Magnesium Questionnaire

Scoring

- 30 (correct)
- 10 (incorrect)
- 20 (possibly correct)

Demographic data for general knowledge questions 1–4 not included in the following tally.

General Magnesium Knowledge

1. a. Yes
2. b. No
3. a. Yes (maximum possible score: 390)
4. a. 350 mg/day; others accepted at lower score: b. 10; c. 20.
5. a. Yes
6. b. No
7. a. Yes
8. b. No; a. 10; c. 20
9. a. Yes
10. d. None of the above; a, 10; b. 10; c. 20
11. a. Yes
12. a. 0.8 mmol/L; b. 10; c. 20
13. a. Yes; b. 10

Clinical Practice

1. a. Yes
2. a. Yes
3. a. Yes
4. a. Yes

Maximum possible score: 120

Appendix S2. Post-Intervention 1-Month Follow-Up Survey With Key

Application of Information to Clinical Practice

1. Have you used the clinical guidelines?
   a. Always
   b. Most of the time
   c. Sometimes
   d. Rarely
2. Now that you have the clinical guidelines, do you feel you have and or will continue to use the **<u>At-a-Glance</u> tool** and **<u>laboratory reference</u>** to identify patients at risk for magnesium deficiency?
   a. Always
   b. Most of the time
   c. Sometimes
   d. Rarely
3. Do you find these clinical guidelines helpful?
   a. Always
   b. Most of the time
   c. Sometimes
   d. Rarely
4. Do you include magnesium deficiency in your differential diagnosis?
   a. Always
   b. Most of the time
   c. Sometimes
   d. Rarely

5 Do you test or intend to test for magnesium deficiency?
   a. Always
   b. Most of the time
   c. Sometimes
   d. Rarely

6. Are you treating patients for magnesium deficiency?
   a. Always
   b. Most of the time
   c. Sometimes
   d. Rarely

Key to the Post-Intervention 1-Month Follow-Up Survey: Application of Information to Clinical Practice

Scoring

- Always: 40
- Most of the Time: 30
- Sometimes: 20
- Rarely: 10

Maximum possible score: 240
